# Research on Prediction of Multiple Degenerative Diseases and Biomarker Screening Based on DNA Methylation

**DOI:** 10.3390/ijms26010313

**Published:** 2025-01-01

**Authors:** Ruoting Tian, Hao Zhang, Chencai Wang, Shengyang Zhou, Li Zhang, Han Wang

**Affiliations:** 1 College of Computer Science and Engineering, Changchun University of Technology, Changchun 130051, China; 2202203074@stu.ccut.edu.cn (R.T.); 20212304@stu.ccut.edu.cn (C.W.); 2202403140@stu.ccut.edu.cn (S.Z.); 2 School of Information Science and Engineering (School of Software), Yanshan University, Qinhuangdao 066000, China; zhanghao2113@stumail.ysu.edu.cn; 3 School of Information Science and Technology, Institute of Computational Biology, Northeast Normal University, Changchun 130117, China; wangh101@nenu.edu.cn

**Keywords:** DNA methylation, degenerative disease, biomarker

## Abstract

The aging process will lead to a gradual functional decline in the human body, and even accelerate a significantly increased risk of degenerative diseases. DNA methylation patterns change markedly with one’s age, serving as a biomarker of biological age and closely linked to the occurrence and progression of age-related diseases. Currently, diagnostic methods for individual degenerative diseases are relatively mature. However, aging often accompanies the onset of multiple degenerative diseases, presenting certain limitations in existing diagnostic models. Additionally, some identified DNA methylation biomarkers are typically applicable to only one or a few types of cancer or diseases, further restricting their utility. We endeavor to screen for biomarkers associated with multiple degenerative diseases from the perspective of aging-related co-morbid mechanisms and to perform multiple degenerative disease diagnoses. In this study, we explored research based on methylation correlations and patterns to investigate shared mechanisms across multiple degenerative diseases, identifying a set of biomarkers associated with them. We validated these biomarkers with biological omics analysis and the prediction of multiple classes of degenerative diseases, screened the biomarkers from 600 to 110 by biological omics analysis, and demonstrated the validity and predictive ability of the screened 110 biomarkers. We propose a disease diagnostic model based on a multi-scale one-dimensional convolutional neural network (MSDCNN) and a multi-class degenerative disease prediction model (ResDegNet). The two models are well trained and tested to accurately diagnose diseases and categorize four types of degenerative diseases. The research identified 110 biomarkers associated with degenerative diseases, providing a foundation for further exploration of age-related degenerative conditions. This work aims to facilitate early diagnosis, the identification of biomarkers, and the development of therapeutic targets for drug interventions.

## 1. Introduction

Aging is a complex biological process characterized by a gradual decline in various bodily functions over time, which significantly increases the risk of developing degenerative diseases, such as cancer, Parkinson’s disease, Alzheimer’s disease, rheumatoid arthritis, and other aging-related conditions [1,2,3]. These degenerative diseases are closely associated with aging, with pathogenesis involving numerous physiological changes and gene regulatory mechanisms [4,5]. In this context, DNA methylation (DNAm) emerges as a key epigenetic modification that plays an essential role in human development and aging [6]. DNAm is involved in regulating gene expression, genomic imprinting, and the silencing of repetitive DNA sequences, among other critical biological processes [7]. Research has demonstrated that DNA methylation patterns undergo significant age-related changes, serving as biomarkers of biological age and correlating with the onset and progression of many age-related diseases, including autoimmune disorders, neurological diseases, and cancers [8]. Utilizing DNA methylation data for diagnosing degenerative diseases and cancers holds significant potential in medical research, as detecting alterations in DNA methylation patterns can help identify early signs of disease [9,10], offering opportunities for timely intervention and treatment. This approach also deepens the understanding of disease mechanisms, prognosis [11], and treatment responses, thereby supporting personalized medicine. Identifying specific DNA methylation sites with biological significance and clinical potential is essential for early screening and diagnosis of diseases [12], discovery of potential biomarkers [13], and exploration of therapeutic targets.

With technological advancements and ongoing exploration, machine learning methods have been widely applied to degenerative disease diagnostics. Nowadays, many researchers use DNA methylation data to study the diagnosis of individual and multiple cancers, as well as neurodegenerative diseases such as Parkinson’s and Alzheimer’s diseases. Hao et al. [14] utilized DNA methylation data to construct a multi-classification system using the Lasso regression method to predict cancer versus normal tissues. Choi et al. [15] introduced meth-SemiCancer, a semi-supervised cancer subtype classification framework based on DNA methylation profiles and built with neural network methods. Park et al. [16] used gene expression and DNA methylation data to predict Alzheimer’s disease through a deep neural network model. Similarly, Amiri et al. [17] successfully predicted rheumatoid arthritis risk by integrating SNP marker information with methylation profiles using a Bayesian regression model. Rahal et al. [18] proposed a method of model ensembling, which combines the weights of several local deep learning models to build a single global model that can accurately diagnose three diseases: breast cancer, lung cancer, and diabetes. Zhang, S et al. [19] compared methylation profiles of BRCA patients, healthy breast biopsies, and blood samples to identify BRCA-specific methylation sites, using a BayesNet model to differentiate BRCA patients from healthy donors.

Currently, many studies focus on identifying biomarkers from DNA methylation data for single diseases, such as specific cancers or degenerative conditions. However, as the prevalence of degenerative diseases and cancers increases with age [4,20], this single-disease approach has limitations in clinical applications. Some DNA methylation markers are effective only for specific cancer types or single degenerative diseases, reducing their generalizability and utility [21]. In contrast, multiple degenerative diseases often share common biological mechanisms, such as aging, cellular dysfunction, and inflammatory responses [22]. Investigating these shared mechanisms provides an opportunity to identify cross-disease biomarkers with broader clinical relevance [23]. Such biomarkers can enhance early screening and detection for multiple diseases, significantly improving the general applicability of diagnostic tools and enabling precision in early diagnosis [24]. Particularly in older populations, who are at a higher risk for multiple degenerative diseases and cancers, biomarkers that are applicable across multiple diseases can support personalized treatment regimens, ultimately improving the precision and effectiveness of medical interventions [25].

In this study, we adopted a novel approach to identify a set of biomarkers associated with multiple degenerative diseases by analyzing aberrant methylation patterns and uncovering DNA methylation features linked to shared mechanisms across these conditions. This cross-disease diagnostic strategy combines machine learning and deep learning methods to account for both nonlinear relationships and synergistic effects among CpG sites. Biomarker loci were rigorously screened using omics analyses, resulting in the identification of 110 biomarker sites associated with multiple degenerative diseases. To validate the utility of these biomarkers, we constructed a multi-class prediction model using deep learning techniques, categorizing neurodegenerative diseases, bone degenerative diseases, breast cancer, and healthy samples. The model demonstrated significant predictive accuracy and robustness, confirming the clinical value of the identified biomarkers. This methodology can be extended in future studies to other age-related degenerative diseases, advancing early diagnosis, biomarker discovery, and therapeutic target identification.

## 2. Results

### 2.1. Disease-Specific Methylation Biomarker Screening

We extracted common CpG sites from three DNA methylation datasets—27 K, 450 K, and 850 K—yielding a total of 12,578 CpG sites. These DNA methylation data were used as input for the random forest model to identify disease biomarkers. During this process, CpG sites with a feature importance value of 0 were removed, resulting in 6438 CpG sites with non-zero importance values. These sites were then ranked in descending order based on their feature importance values, allowing us to retain only the CpG sites most relevant to disease and cancer prediction. This filtering step is essential for isolating CpG sites highly associated with specific diseases, as it removes those with minimal predictive value.

To evaluate the model’s performance, we used four metrics: accuracy, precision, recall, and MCC (Matthews Correlation Coefficient). Table 1 presents the results for models with 12,578 and 6438 CpG sites. After removing features with zero importance scores, the model with 6438 CpG sites achieved slightly higher MCC (0.962) and accuracy (0.987) than the model with 12,578 CpG sites (MCC: 0.958; accuracy: 0.986). Although the precision (0.968) and recall (0.971) values for the 6438-feature model were marginally lower than those for the 12,578-feature model (precision: 0.991; recall: 0.992), the model maintained high overall accuracy. This indicates that the removal of low-importance features had minimal impact on the prediction of diseases and cancer, confirming the robustness of the selected CpG sites.

### 2.2. Valuable Disease-Specific Methylation Biomarker Screening

While machine learning methods account for the nonlinear relationships between CpG sites and degenerative diseases, we further employed deep learning techniques in the second phase to explore the synergistic effects of CpG sites on degenerative diseases and cancer, as well as to capture both local and global features of CpG sites. This approach allows for a comprehensive understanding of the complex associations between CpG site interactions and disease outcomes. By using a multi-scale one-dimensional convolutional neural network, we were able to capture CpG site interactions at different scales, revealing both localized and global features that contribute to degenerative diseases and cancer.

To optimize the number of CpG sites for predicting degenerative diseases and cancer, we began with the 6438 CpG sites selected from the initial random forest screening. Using an iterative approach, we started with 150 CpG sites as model input, gradually increasing this number in increments of 150 until reaching a total of 6348 CpG sites. This stepwise approach allowed us to evaluate the impact of different CpG site combinations on predictive accuracy, enabling the identification of the most influential set of CpG sites for optimal disease prediction outcomes.

**Figure 1:**
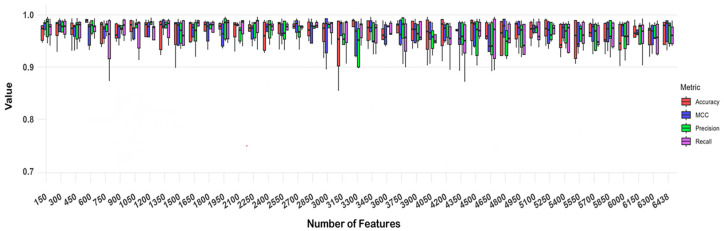
Box plots of evaluation metrics for different feature counts under five-fold cross-validation. The figure presents box plots of evaluation metrics for various feature counts across each fold of cross-validation. As the number of CpG sites used as model input increased from 150 to 6438, the evaluation metrics gradually stabilized.

To assess model performance reliably and mitigate evaluation bias due to overfitting or uneven data distribution, we used five-fold cross-validation. We evaluated the model using four metrics: accuracy, precision, recall and MCC (Matthews Correlation Coefficient), and analyzed the results across each fold of the cross-validation. Figure 1 presents box plots of these metrics across different feature counts for each fold of the cross-validation. We observed that as the number of CpG sites increased from 150 to 6438, the evaluation metrics stabilized after five-fold cross-validation. Thus, we averaged the results across the five folds to assess the overall performance for each feature count.

Following the principle of selecting the minimum number of CpG sites needed to achieve optimal results, we ultimately chose 600 CpG sites for further analysis. As shown in Table 2, the model’s performance with 600 CpG sites yielded an accuracy of 0.988, precision of 0.952, and MCC of 0.959—slightly surpassing the results obtained with 6438 CpG sites (accuracy: 0.986; precision: 0.946; MCC: 0.956). Although the recall for the 600 CpG sites (0.983) was marginally lower than that for the 6438 CpG sites (recall: 0.985), this difference was minimal, maintaining a high level of predictive accuracy. These results suggest that the synergistic interactions among the selected 600 CpG sites provide a more accurate prediction of diseases and cancers than those among the full set of 6438 CpG sites.

### 2.3. Degenerative Disease-Specific Biomarkers Identifying

#### 2.3.1. Enrichment Analysis

**Figure 2:**
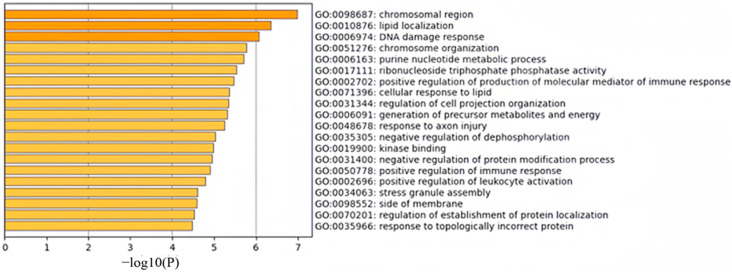
Visualization of the top 20 clusters from GO functional enrichment analysis. GO terms with a *p*-value < 0.01, a minimum count of 3, and an enrichment factor >1.5 were selected and grouped into clusters based on the similarity of their members.

We mapped the selected 600 CpG sites to their corresponding genes and performed Gene Ontology (GO) functional enrichment analysis and Kyoto Encyclopedia of Genes and Genomes (KEGG) pathway enrichment analysis on these genes. Figure 2 displays the top 20 clusters from the GO functional enrichment analysis, including 16 Biological Process (BP) terms, 2 Cellular Component (CC) terms, and 2 Molecular Function (MF) terms. The distribution indicates that the BP category contains more substantial information.

In terms of biological processes, a significant number of CpG sites were enriched in lipid localization, DNA damage response, chromatin organization, purine nucleotide metabolism, positive regulation of immune response mediator production, cellular response to lipids, regulation of cell projection organization, precursor metabolite and energy generation, response to axon injury, and negative regulation of dephosphorylation. Cellular component enrichment was mainly focused on chromosomal regions and membrane compartments, while molecular functions showed significant enrichment in ribonucleoside triphosphate phosphatase activity and kinase binding. Overall, this gene set appears to be involved in cellular structures such as chromosomal regions and cell membranes. Changes in chromosomal regions may relate to chromosome abnormalities [26] and DNA replication [27], whereas membrane components may be associated with cell membrane organization and function, as well as processes like cell signaling and substance exchange [28].

**Figure 3:**
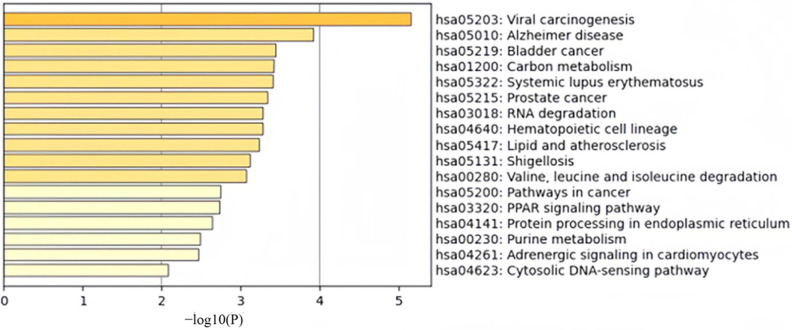
Visualization of KEGG pathway enrichment analysis results based on *p*-values. Seventeen KEGG pathways were identified, including Viral Carcinogenesis, Alzheimer’s Disease, Bladder Cancer, Carbon Metabolism, Systemic Lupus Erythematosus, Prostate Cancer, RNA Degradation, Hematopoietic Cell Lineage, Lipid and Atherosclerosis, Shigellosis, Valine, Leucine, and Isoleucine Degradation, Cancer Pathways, PPAR Signaling Pathway, Protein Processing in the Endoplasmic Reticulum, Purine Metabolism, Adrenergic Signaling in Cardiomyocytes, and Cytosolic DNA-Sensing Pathway.

For the KEGG pathway enrichment analysis, a total of 17 pathways were identified among the selected 600 CpG sites, as shown in Figure 3. Specifically, pathways such as viral carcinogenesis, Alzheimer’s disease, bladder cancer, prostate cancer, and systemic lupus erythematosus are associated with various cancers and neurological disorders, revealing potential key signaling pathways involved in the pathogenesis of these diseases. Additionally, pathways like carbon metabolism, RNA degradation, valine, leucine and isoleucine degradation, and purine metabolism are involved in cellular metabolic processes, which may be related to metabolic disorders and disease progression [29]. Enrichment analysis also indicates that pathways such as hematopoietic cell lineage, lipid and atherosclerosis, protein processing in the endoplasmic reticulum, PPAR signaling pathway, adrenergic signaling in cardiomyocytes, and cytosolic DNA-sensing pathway participate in cellular signal transduction, protein synthesis, and cellular stress responses, among other biological functions [30,31].

#### 2.3.2. Find Hub Genes

To construct the PPI network, we uploaded the genes associated with the 600 selected CpG sites to the STRING tool. The enrichment analysis of the resulting PPI network revealed 31 GO terms, 3 KEGG pathways, 224 Reactome pathways, 9 UniProt Keywords, and 1 WikiPathway. The identified WikiPathway represents an integrative cancer pathway, with a strength index of 0.82, involving 10 key genes: *MSH2, RB1, TP53, CDKN2B, PLK1, MMP1, NOXA1, BAD, BRCA1*, and *AKT1*.

**Figure 4:**
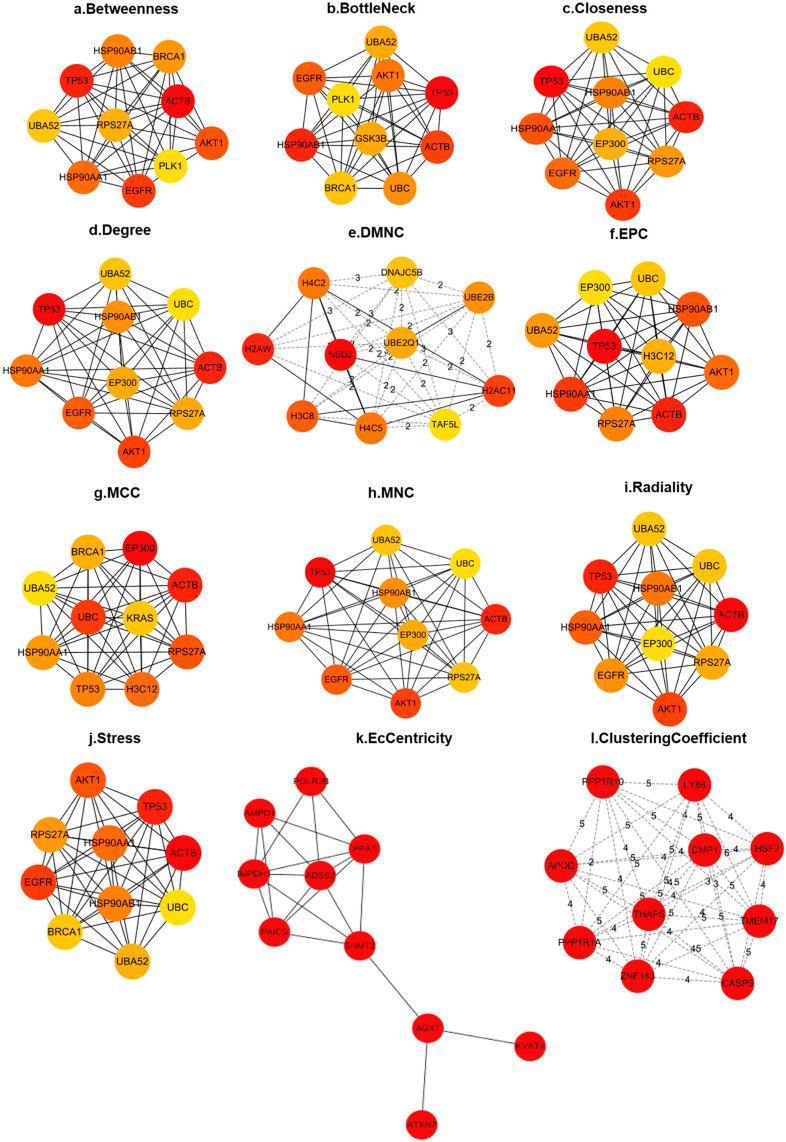
Key subnetworks composed of hub genes identified by different algorithms. Genes within the network were evaluated and scored using different algorithms to obtain a critical subnetwork of hub genes. Among the results of the 12 algorithms (**a**–**l**), common hub genes that consistently scored high were screened.

To further analyze the constructed PPI network, we employed the CytoHubba plugin in Cytoscape software version 3.10.1to evaluate and score genes within the network using various algorithms. This approach enabled the identification of key subnetworks composed of hub genes. Figure 4 presents these key subnetworks based on different algorithmic analyses. Across the results of twelve algorithms, the common hub genes with consistently high scores include *ACTB, TP53, AKT1, EGFR, HSP90AA1, HSP90AB1, RPS27A, UBA52, EP300, BRCA1*, and *UBC*.

#### 2.3.3. Recognize Key Subnetworks

**Figure 5:**
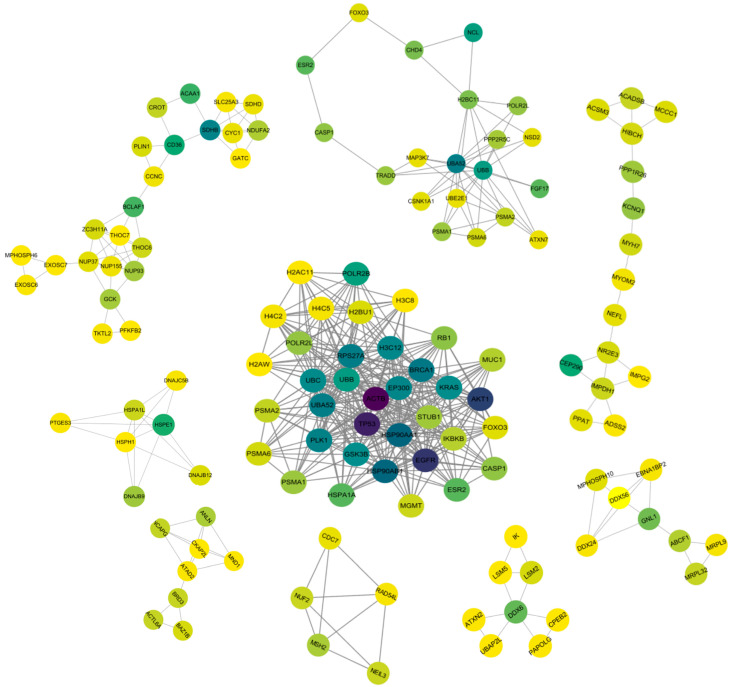
The nine subnetworks identified from PPI network analysis using the MCODE plugin. Node colors indicate interactivity and importance, with darker nodes in each subnetwork representing a higher number of interacting edges and greater importance.

The MCODE plugin identifies “hot spots” within the PPI network, which are clusters of protein or gene nodes with high density and strong interactions. These clusters are often biologically significant, playing key roles in processes such as signaling pathways and protein interaction complexes. Using the MCODE plugin to analyze the PPI network, we identified and isolated 16 regions (subnetworks) closely associated with the network. Table 3 presents detailed information on these 16 subnetworks. For further analysis, we selected nine subnetworks based on their high scores and the number of nodes they contain, specifically the subnetworks ranked 1st, 2nd, 3rd, 4th, 5th, 6th, 7th, 12th, and 16th. Figure 5 illustrates these nine subnetworks identified by the MCODE plugin from the PPI network.

The first subnet (a) consists of 36 genes and 342 interaction edges. The pathways associated with this PPI network primarily involve Shigellosis, Prostate Cancer, Kaposi’s Sarcoma-associated Herpesvirus Infection, Neutrophil Extracellular Trap Formation, Viral Carcinogenesis, Fluid Shear Stress and Atherosclerosis, Huntington’s Disease, Necroptosis, Platinum Drug Resistance, Cushing Syndrome, and Transcriptional Dysregulation in Cancer. High-scoring common hub genes identified by the CytoHubba plugin—such as *ACTB, TP53, AKT1, EGFR, HSP90AA1, HSP90AB1, RPS27A, UBA52, EP300, BRCA1*, and *UBC*—are all present in this top-ranked subnet.

The third subnet (c) includes 20 genes and 47 interaction edges. Pathways in this network are largely associated with Shigellosis, Neurodegenerative Pathways in Multiple Diseases, Pathogenic Escherichia coli Infection, Human Papillomavirus Infection, and the AMPK Signaling Pathway.

The fifth subnet (e) contains 24 genes and 49 interaction edges. The pathways involved in this PPI network mainly include Amyotrophic Lateral Sclerosis, Nuclear Transport, PPAR Signaling Pathway, and RNA Degradation.

The second (b), fourth (d), sixth (f), seventh (g), and eighth (h) subnetworks primarily involve pathways related to Protein Processing in the Endoplasmic Reticulum, DNA Repair, Chromosome Organization, rRNA Processing, and RNA Degradation. The sixteenth subnet (i) is mainly associated with pathways involving α-Amino Acid Metabolic Process, Purine Ribonucleotide Monophosphate Biosynthetic Process, Sensory Organ Development, Muscle Contraction, and Hormone Response.

The nine selected key subnetworks involve a total of 123 genes, corresponding to 382 CpG sites. Among these, 110 CpG sites fall within the top 600 CpG sites previously identified, and are associated with the development of degenerative diseases, cellular biology, metabolic processes, and sensory organ development.

#### 2.3.4. Cluster Analysis of CpG Sites

To cluster the DNA methylation data corresponding to these 110 CpG sites, we applied the Louvain community detection method. This approach assigns samples to different communities (or clusters) based on the graph’s structure and similarity relationships among nodes. To build the graph’s structure and establish node similarities, we first calculated the cosine similarity between each sample (node) and all other nodes, selecting the K nearest neighbors for each node. These similarity relationships were then added to the graph, with nodes representing samples and edges indicating their similarity relationships. The resulting communities represent clusters of highly similar samples, each potentially representing a distinct category.

**Figure 6:**
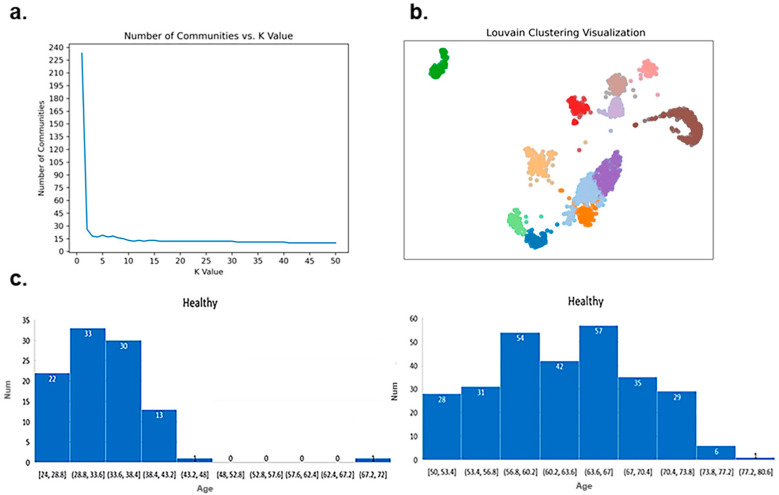
Louvain community clustering analysis for DNA methylation data of 110 CpG sites. (**a**) The trend in the number of communities as the K value varies, with stabilization observed at K = 16. (**b**) Clustering results from Louvain community detection, showing 12 communities when K = 16. (**c**) Age distribution histograms for the two communities of healthy samples, with mean ages of 33.74 years and 62.38 years, respectively.

To determine the optimal value of K (the number of nearest neighbors), we limited K to values between 1 and 50, observing how the number of communities stabilized as K increased. Experimental results indicated that when K is set to 16, the number of communities stabilizes with minimal variation, suggesting that the community structure reaches a steady state within this range. Figure 6a shows the trend in the number of communities as K varies. When K is set to 16, the number of communities is 12, indicating a stable clustering configuration.

Figure 6b presents the clustering results obtained using the Louvain community detection algorithm. Among the twelve identified communities, the following associations were observed: Progressive supranuclear palsy and frontotemporal dementia were grouped into the same community, while frontotemporal dementia and Alzheimer’s disease clustered together in a separate group. Alzheimer’s disease and Parkinson’s disease each formed distinct individual communities. Rheumatoid arthritis was divided into two separate communities, and breast cancer samples were distributed across four distinct communities. Additionally, healthy samples were split into two communities, with a notable age difference between them. Figure 6c illustrates the age distribution histograms for these two healthy communities, with mean ages of 33.74 years and 62.38 years, respectively.

### 2.4. Multi-Class Degenerative Disease Prediction

Through the integration of machine learning, deep learning, and bioinformatics analysis, we identified a set of CpG sites associated with multiple degenerative diseases. To further investigate the relationships between these CpG sites and various types of degenerative diseases, we developed a multi-class prediction model named ResDegNet. In this model, the DNA methylation expression matrix data corresponding to the selected CpG sites are used as input, while the four categories—neurological degenerative diseases, bone degenerative diseases, breast cancer, and healthy samples—serve as output labels.

The input data were split into training and testing sets with an 8:2 ratio. The training set was used for model training and five-fold cross-validation, while the testing set served for independent validation, allowing us to assess the model’s generalization ability and performance. Four evaluation metrics—accuracy, precision, recall, and F1 Score—were used to evaluate the model’s effectiveness. Table 4 presents the results of these metrics across each fold of the cross-validation. On the independent test set, the multi-class degenerative disease prediction model achieved an accuracy of 0.984, precision of 0.984, recall of 0.985, and F1 score of 0.984.

These results demonstrate that the 110 selected CpG biomarkers have strong predictive power for accurately distinguishing between neurodegenerative diseases, bone degenerative diseases, breast cancer, and healthy samples.

To further assess whether ResDegNet effectively learns the methylation patterns associated with the four sample categories—neurodegenerative diseases, bone degenerative diseases, breast cancer, and healthy samples—we conducted a clustering analysis using the global feature vectors generated by the Global Average Pooling layer. These global feature vectors offer a comprehensive representation of the entire feature map, encapsulating overall information about the methylation patterns. By clustering these global feature vectors, we can evaluate whether the biomarkers identified for degenerative diseases can reliably distinguish among the four categories.

**Figure 7:**
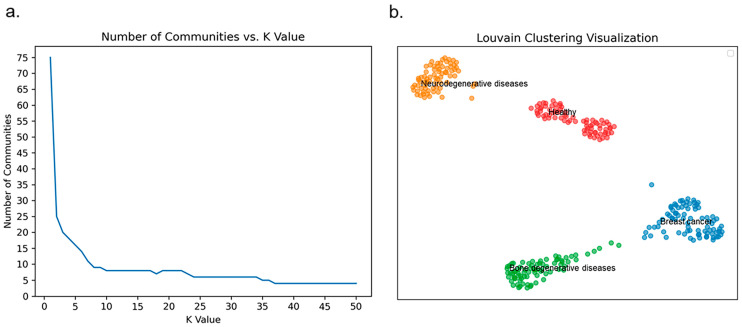
Louvain community clustering analysis using global feature vectors from the Global Average Pooling Layer. (**a**) The trend in the number of communities as the K value changes, showing stabilization at four communities as K increases from 37 to 50. (**b**) Clustering results from the Louvain community detection method, with four communities identified when K is set to 37.

For the clustering process, we employed the Louvain community detection method on the global feature vectors. Experimental results indicated that as the value of K increased from 37 to 50, the number of communities stabilized at four, suggesting a stable clustering configuration within this range. Figure 7a illustrates the trend in the number of communities as K varies. When K is set to 37, the number of communities stabilizes at four, with each community containing 81, 79, 73, and 78 nodes, respectively. In the independent test set, the sample counts for neurodegenerative diseases, bone degenerative diseases, breast cancer, and healthy samples are 82, 78, 74, and 77, respectively, closely matching the distribution within the clustered communities.

To further verify the correspondence between the number of nodes in each community and the number of samples in each category, we examined both the community membership of each node and the actual sample categories. The results are as follows:

The neurodegenerative diseases community contains 81 nodes; 80 nodes were correctly classified as neurodegenerative diseases, while 1 node actually belongs to the bone degenerative disease category. According to ResDegNet’s predictions, this node has a 0.795 probability of being classified as a neurodegenerative disease and a 0.2048 probability of being classified as a bone degenerative disease, indicating a strong but not definitive classification.

The bone degenerative diseases community contains 79 nodes; 77 nodes were correctly classified as bone degenerative diseases, while 2 nodes actually belong to the neurodegenerative disease category. For these two misclassified nodes, ResDegNet assigned one a 0.03 probability of being a neurodegenerative disease and a 0.9968 probability of being a bone degenerative disease, while the other was assigned a 0.8578 probability of being a neurodegenerative disease and a 0.142 probability of being a bone degenerative disease.

The breast cancer community contains 73 nodes, with all 73 nodes correctly classified as breast cancer, demonstrating ResDegNet’s high accuracy in this category.

The healthy community contains 78 nodes; 77 nodes were correctly classified as healthy, while 1 node actually belongs to the breast cancer category. However, ResDegNet’s prediction for this node indicated a 0.058 probability of breast cancer and a 0.94 probability of being healthy, reflecting a high confidence in the healthy classification.

Figure 7b displays the clustering results using the Louvain community detection method. Overall, the set of 110 biomarkers identified for various degenerative diseases has demonstrated significant effectiveness in distinguishing among multiple degenerative disease categories. Furthermore, ResDegNet showed a high level of discriminatory power in predicting these disease categories, validating its robust predictive performance.

To evaluate the impact of each feature on model predictions and determine the relative importance of each CpG site within the model, we conducted SHAP (SHapley Additive exPlanations) analysis [32]. This analysis allowed us to measure the influence of methylation patterns at specific CpG sites on predicting neurodegenerative diseases, bone degenerative diseases, breast cancer, and healthy states. We calculated the average SHAP value for each CpG site across all samples in the training set, reflecting the overall importance of each CpG site in model predictions.

**Figure 8:**
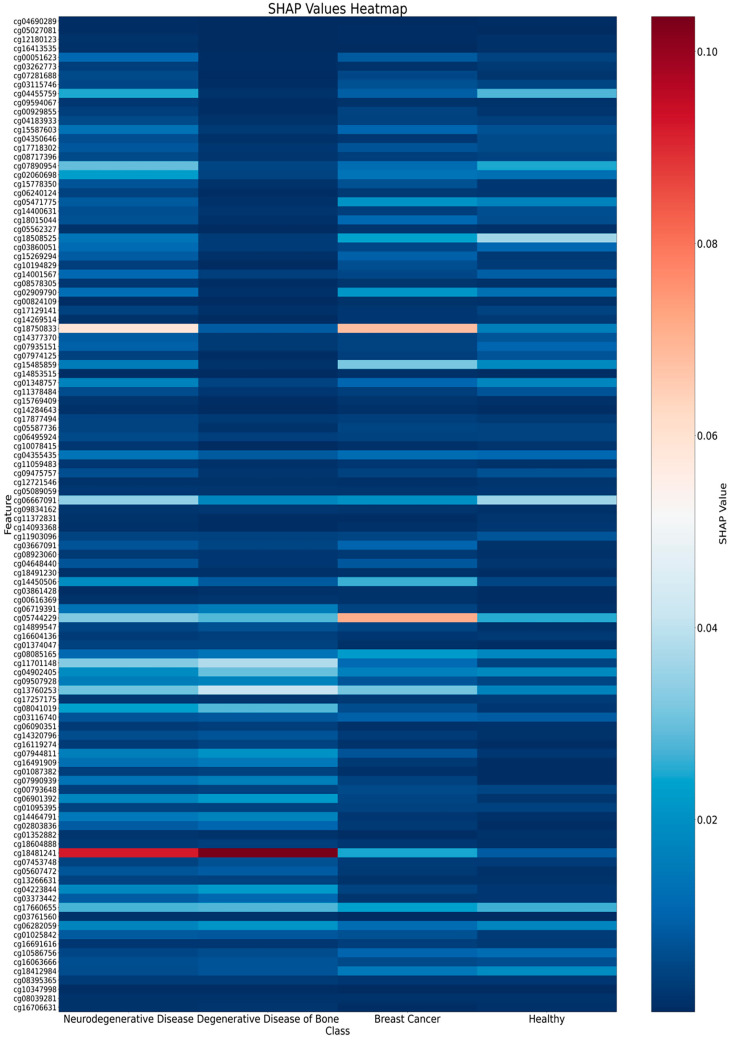
Heatmap of SHAP values for each CpG site across different categories. The SHAP value in the graph is the amount of “contribution” each biomarker makes to the prediction, indicating the degree of influence the biomarker has on the predicted results of each of the four categories. The larger the SHAP value of the biomarker, the greater the influence, which tends to be in the red color. Conversely, lower values tend to be blue. The heatmap highlights the importance of specific CpG sites in predicting each category. Notably, cg18481241 plays a significant role in predicting neurodegenerative and bone degenerative diseases, cg05744229 is crucial for breast cancer prediction, and cg18750833 is important for predicting both neurodegenerative diseases and breast cancer.

Each CpG site’s SHAP values are presented as four values, corresponding to its importance in predicting neurodegenerative diseases, bone degenerative diseases, breast cancer, and healthy states. Figure 8 displays a heatmap of SHAP values for each CpG site across these categories. The heatmap reveals that cg18481241 has a significant impact on predicting neurodegenerative and bone degenerative diseases, cg05744229 plays a crucial role in predicting breast cancer, and cg18750833 is influential in predicting both neurodegenerative diseases and breast cancer.

## 3. Discussions

After histological analysis, *ACTB, TP53, AKT1, EGFR, HSP90AA1, HSP90AB1, RPS27A, UBA52, EP300, BRCA1*, and *UBC* were identified as key genes in the PPI net-work and crucial components of the key subnetworks. Among these, *ACTB* and *TP53* are closely associated with various cancers, playing pivotal roles in tumorigenesis and cellular regulation [33,34]. *AKT1* regulates cell proliferation, survival, and metabolism [35], while mutations in *EGFR* increase lung cancer sensitivity to tyrosine kinase inhibitor therapy [36]. *HSP90AA1* and *HSP90AB1* facilitate oncogene addiction, critical for cancer progression [37]. *RPS27A* is linked to colorectal cancer, hepatocellular carcinoma, chronic granulocytic leukemia, and renal cancer, with poor prognostic implications [38]. *UBA52* plays a vital role in *HSP90* ubiquitination and neurodegenerative signaling in Parkinson’s disease [39]. *EP300*, a histone acetyltransferase, regulates cell proliferation by mediating *TP53* acetylation, essential for DNA damage response [40]. *BRCA1* is associated with early-onset breast cancer and familial breast cancer syndrome [41]. Lastly, *UBC* encodes ubiquitin, responding robustly to cellular stressors such as ultraviolet irradiation, oxidative stress, and translational damage [42].

According to the human genome reference sequence annotation (hg38), cg18481241 maps to the *PPP1R26P1* and *RB1* genes. *RB1*, a well-known tumor suppressor, encodes the retinoblastoma protein (pRb) [43]. cg05744229 is associated with the *MYH7* gene, which encodes β-myosin heavy chain (Myosin-7), a critical protein for muscle contraction. Mutations in *MYH7* are linked to muscular dystrophy and hypertrophic cardiomyopathy [44]. cg18750833 maps to both the *MCEE* and *MPHOSPH10* genes. *MCEE* encodes methionine-ethionine carboxy-lyase, essential for sulfur metabolism [45], while *MPHOSPH10* encodes M-phase phosphoprotein 10, implicated in ribosome biogenesis and associated with Parkinson’s disease [46]. Additionally, we observed a high prevalence of degenerative bone diseases in patients with Parkinson’s and Alzheimer’s disease, suggesting potential co-morbidities.

Regression and machine learning methods are widely used to identify disease-associated biomarkers and enable disease diagnosis. For example, van Breugel et al. [47] utilized an Elastic Net model to classify allergic diseases in Dutch children based on nasal CpG methylation sites. Li et al. [48] implemented supervised machine learning algorithms to classify blood samples as malignant or non-malignant based on tissue-specific methylation. Ren et al. [49] identified differentially methylated sites using DNA methylation data and applied random forest models to select diagnostic biomarkers for Alzheimer’s disease. While most studies use methods like Elastic Net and Lasso regression for biomarker screening followed by targeted disease diagnosis, few integrate multiple approaches for enhanced screening. Lin et al. [50] combined feature reduction using the coefficient of variation with Elastic Net to select important features and constructed a neural network to classify cancer types effectively.

Our study combines machine learning and deep learning approaches to screen biomarkers associated with degenerative diseases, offering unique advantages. These methods capture nonlinear relationships and synergistic effects of CpG sites, enabling the identification of biomarkers with cross-disease relevance. While most identified biomarkers are specific to single diseases, our approach identifies a set of shared biomarkers, addressing limitations of current methods. This facilitates exploring common biological mechanisms, uncovering disease connections, and promoting early diagnosis, precision therapy, and personalized medicine across disease categories.

Despite the success in identifying 110 biomarkers associated with multiple degenerative diseases and validating their utility, limitations remain. Our analysis relies on DNA methylation data, and future studies should expand sample diversity, integrate DNA methylation with histological and longitudinal data, and explore biomarker performance across different populations. Recent studies, such as Park et al. [16], demonstrate the potential of integrating multi-omics data to enhance degenerative disease predictions. Combining such insights with our approach could refine biomarker screening strategies and support targeted treatments for a broader spectrum of degenerative diseases.

DNA methylation-based biomarkers play a crucial role in diagnosing degenerative diseases and cancers, facilitating early detection and advancing understanding of disease mechanisms during aging. Future research should focus on using similar screening strategies to identify biomarkers for early screening in high-risk populations. These efforts would support personalized treatments and interventions. Additionally, studying methylation patterns could help address potential co-morbidities, driving the discovery of novel therapeutic targets and innovative approaches for degenerative disease management. These biomarkers provide the foundation for precise diagnostic tools and targeted therapies, promoting the widespread adoption of personalized medicine.

## 4. Materials and Methods

**Figure 9:**
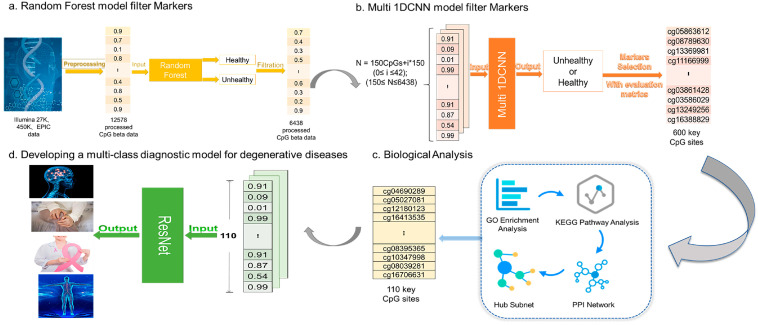
Overall technical approach. The proposed technical approach includes four parts. Firstly, filtering biomarkers with zero feature importance using the random forest method. Secondly, further selecting biomarkers related to multiple degenerative diseases using deep learning methods. Thirdly, analyzing the selected biomarkers using omics analysis methods. Finally, constructing a multi-class degenerative disease prediction model for validation.

In this section, we describe the DNA methylation dataset and outline the methods used to select a representative set of biomarkers and construct a multi-class degenerative disease prediction model. As shown in Figure 9, our technical framework comprises four key steps: first, filtering biomarkers using the random forest method; second, selecting an optimal set of biomarkers through deep learning techniques; third, performing omics analysis; and finally, constructing a multi-class prediction model for degenerative diseases.

### 4.1. Datasets

We obtained 142 publicly available DNA methylation datasets from the Gene Expression Omnibus (GEO) database [51] https://www.ncbi.nlm.nih.gov/geo/ (accessed on 27 September 2023), covering platforms including Illumina Infinium HumanMethylation27K, Illumina Infinium HumanMethylation450K, and Illumina Infinium MethylationEPIC [52].

**Figure 10:**
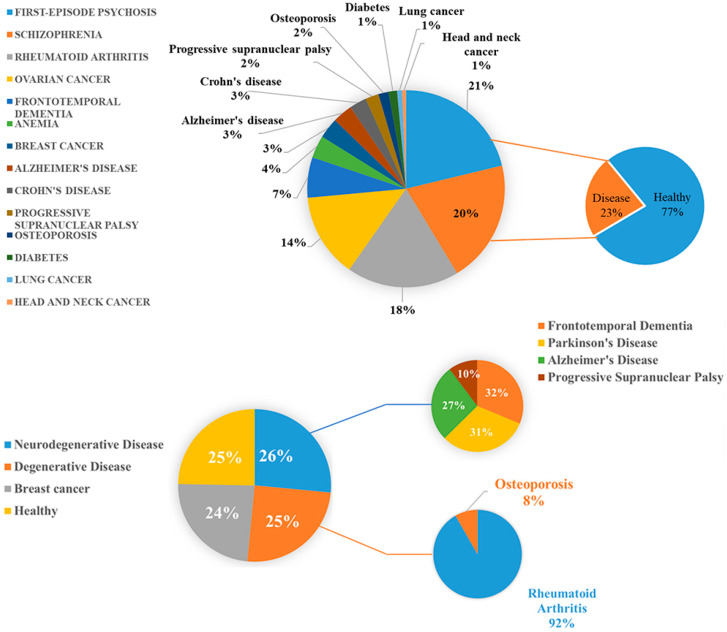
Pie chart of the dataset distribution. The DNA methylation data used for screening a representative set of biomarkers. The dataset includes information on the proportion of healthy and disease samples, with the disease samples further comprising information on the proportion of corresponding disease samples. DNA methylation data for multi-class degenerative disease prediction model. This figure illustrates the four categories of DNA methylation data used to construct the multi-class degenerative disease prediction model, showing the sample proportions for each category, and the breakdown of specific diseases and their sample proportions within each category.

To select a representative set of biomarkers, we focused on samples derived from blood tissues, ultimately retaining 39 datasets for further analysis. These datasets were categorized into two groups: healthy and disease. In the disease group, we excluded samples that were confirmed healthy, retaining only those diagnosed with disease. Figure 10 illustrates the proportion of healthy and disease samples. We divided these samples into a training set (80%, *n* = 6667) and a testing set (20%, *n* = 1668). We labeled healthy samples as 0 and disease samples as 1 for subsequent disease diagnosis and risk prediction.

To construct the multi-class degenerative disease prediction model, we utilized 15 DNA methylation datasets, in which samples were categorized into four classes: neurodegenerative diseases, bone-related degenerative diseases, breast cancer, and healthy. To ensure that the model could effectively learn and represent the characteristics of each category during training and evaluation, we prioritized balancing the sample sizes across classes. The neurodegenerative disease class included 409 samples, the bone-related degenerative disease class included 386 samples, the breast cancer class included 367 samples, and the healthy class included 381 samples. As illustrated in Figure 10, the figure presents the distribution of samples across the four classes, along with the breakdown of specific diseases and the proportion of samples within each category.

For all the samples, we randomly allocated 80% to training (*n* = 1232) and 20% to independent testing (*n* = 311). During this process, we assigned unique labels for each category to facilitate multi-class degenerative disease diagnosis and analysis: 1000 for neurodegenerative diseases, 0100 for bone-related degenerative diseases, 0010 for breast cancer, and 0001 for healthy. This labeling scheme supports the accurate classification of each disease type and enables comprehensive model evaluation.

### 4.2. Screening Disease-Related Biomarkers

**Figure 11:**
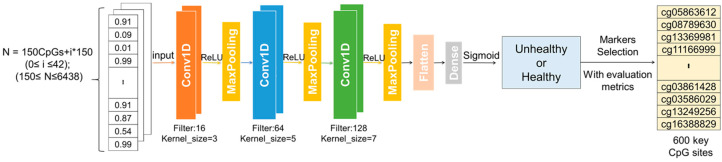
MSDCNN disease diagnosis model based on multi-scale one-dimensional convolutional neural network. The MSDCNN model is designed with three scales, featuring one-dimensional convolutional layers with kernel sizes of 3, 5, and 7, respectively. After every two convolutional layers, a pooling layer is introduced to reduce dimensionality and enhance feature selection. The ReLU activation function is applied to each convolutional layer. A Flatten layer then converts the multidimensional output into a one-dimensional vector, followed by a fully connected layer. A Sigmoid activation function is applied in the final layer to constrain the output to a range between 0 and 1, facilitating binary classification.

We developed the disease diagnosis model using a multi-scale one-dimensional convolutional neural network (MSDCNN). Figure 11 illustrates the structure of the MSDCNN, which incorporates three scales with convolutional kernel sizes of 3, 5 and 7. Smaller convolutional kernels capture local fine-grained features, while larger kernels progressively capture broader patterns, enabling the model to learn more abstract and global features. To further enhance feature extraction, a pooling layer is added after every two one-dimensional convolutional layers, and the ReLU activation function is applied to each convolutional layer.

After the convolutional layers, a Flatten layer converts the multidimensional output data into a one-dimensional vector, followed by a Dense layer. Since our model is designed for binary classification tasks, we set the output layer to have a single neuron, using a Sigmoid activation function to ensure output values between 0 and 1. The model is compiled with the Adam optimizer, and binary cross-entropy is chosen as the loss function.

To determine the model’s final output, we apply a thresholding approach. The output values between 0 and 1 are evaluated against an optimal threshold: values above the threshold are rounded to 1, and those below are rounded to 0. To select this optimal threshold, we analyze the ROC curve and maximize the area under the curve (AUC) by identifying the threshold where the difference between the true positive rate and the false positive rate is greatest. This threshold selection process ensures maximum classification accuracy.

### 4.3. Biological Omics Analysis

#### 4.3.1. Enrichment Analysis

To elucidate the associations between DNA methylation and various biological aspects, such as gene function, expression regulation, and related diseases, and to gain deeper insights into biological processes and disease mechanisms, we performed enrichment analysis by mapping CpG sites to corresponding genes. Specifically, we utilized Gene Ontology (GO) enrichment analysis [53] and Kyoto Encyclopedia of Genes and Genomes (KEGG) enrichment analysis [54] to uncover functional characteristics and signaling pathways within gene sets.

MetaScape is a tool for bioinformatics and biological data analysis, primarily used for functional enrichment analysis and pathway analysis [55]. We mapped selected CpG sites to genes, and performed GO functional enrichment analysis and KEGG pathway enrichment analysis on the gene lists by MetaScape, as a way to determine whether these genes are involved in degenerative disease biological processes and cellular metabolic processes, and to determine the degree of enrichment in cellular components and molecular functions. This helps to gain a deeper understanding of the relationship between the genome and related biological functions, and the mechanisms of functional regulation and action in biological processes.

#### 4.3.2. Protein–Protein Interactions

String is a bioinformatics tool primarily used for analyzing protein–protein interaction networks [56]. We mapped selected CpG sites to genes. Using these gene lists, String constructs protein–protein interaction networks by computationally predicting interactions, integrating known experimental data and literature reports. This approach reveals protein–protein interactions and builds comprehensive networks. It helps to understand the functional regulation and signaling networks of proteins in the cell.

#### 4.3.3. Key Subnetworks and Hub Genes

Through the CytoHubba plugin [57] and MCODE plugin [58] in Cytoscape software version 3.10.1 [59], hub genes were selected and key subnetworks were discovered in the constructed PPI network. The constructed PPI network was imported into Cytoscape software, and multiple algorithms corresponding to different plugins were used to select the pivotal genes and key subnetworks of the whole PPI network, so as to find the genes and subnetworks that play key roles in the process of degenerative diseases.

### 4.4. Classification Model for Degenerative Diseases

**Figure 12:**
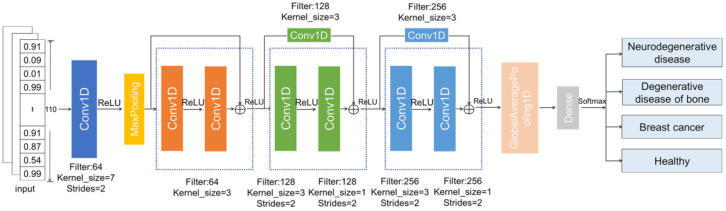
Network architecture of the multi-class degenerative disease prediction model, ResDegNet. The ResDegNet model begins with a one-dimensional convolutional layer for feature extraction, followed by a max pooling layer to downsample the feature maps. Three residual blocks are then introduced, with each convolutional layer within these blocks employing the ReLU activation function. A Global Average Pooling layer is applied next, reducing the dimensionality of the feature maps by averaging all elements within each channel, producing a single vector as the model’s output. In the final output layer, a fully connected (Dense) layer with 4 neurons uses the SoftMax activation function to yield the probability distribution across the four classes.

Figure 12 provides an overview of the architecture of our multi-class degenerative disease prediction model, ResDegNet, which is based on a residual network (ResNet). In this model, we built a prediction framework to classify multiple types of degenerative diseases. Initially, a one-dimensional convolutional layer was employed for feature extraction, followed by a MaxPooling layer to reduce the dimensionality of the feature maps.

The core of ResDegNet consists of three residual blocks. Each convolutional layer within these blocks utilizes the ReLU activation function. The first residual block comprises two convolutional layers, with an Add() operation between them to sum the input and output, preserving information flow. The second and third residual blocks each contain three convolutional layers with varying strides to progressively reduce the feature map dimensions while increasing the number of channels, thereby enhancing the network’s capacity to represent complex features.

A Global Average Pooling layer is applied after the residual blocks, reducing the dimensionality of the feature maps by averaging all elements within each channel, resulting in a single vector output. In the final output layer, a Dense layer with 4 neurons and a SoftMax activation function provides the probability distribution across the four categories.

The model was compiled using the Adam optimizer, with categorical cross-entropy as the loss function. To ensure robust performance assessment and minimize evaluation bias due to overfitting or imbalanced data distribution, we employed five-fold cross-validation for model evaluation.

## 5. Conclusions

This study developed a research approach based on methylation reactions and patterns to explore shared mechanisms underlying various degenerative diseases, ultimately identifying a set of biomarkers associated with multiple degenerative conditions.

Initially, we employed the random forest method for disease diagnosis to filter out CpG sites with zero feature importance. Next, we applied a multi-scale one-dimensional convolutional neural network to analyze the synergistic effects of the selected CpG sites across different degenerative diseases, thereby identifying key biomarkers strongly associated with disease prediction.

We further conducted functional, pathway, and interaction analyses on these biomarkers using omics analysis techniques. This process not only enabled the identification of critical biomarkers important for cancer and degenerative disease diagnosis, but also provided insights into their biological functions.

Finally, we constructed a multi-class degenerative disease diagnostic model using deep learning to validate the predictive power of these biomarkers to distinguish between neurodegenerative diseases, bone degenerative diseases, breast cancer, and healthy states. To ensure balanced samples across categories, we optimized the sample proportions for cancer and neurodegenerative disease, thus enhancing the model’s stability and reliability. Despite limitations in sample size, the model demonstrated strong predictive performance in testing.

In summary, this study provides a potential set of methylation biomarkers for multiple degenerative diseases, laying a foundation for early diagnosis, personalized treatment, and targeted intervention. Future research will aim to expand the dataset to improve the model’s generalization ability, while continuing to investigate early detection, biomarker screening, and drug target development for aging-related degenerative diseases.

## Figures and Tables

**Table 1:** Performance metrics for models with 12,578 and 6438 CpG sites. Bold text indicates that the indicator is advantageous.

NUM	Accuracy	Precision	Recall	MCC
12,578	0.986	**0.991**	**0.992**	0.958
6438	**0.987**	0.968	0.971	**0.962**

**Table 2:** Performance metrics for models with 600 and 6438 CpG sites. Bold text indicates that the indicator is advantageous.

NUM	Accuracy	Precision	Recall	MCC
6438	0.986	0.946	**0.985**	0.956
600	**0.988**	**0.952**	0.983	**0.959**

**Table 3:** Detailed information on the 16 identified regions (subnetworks).

Cluster	Score	Nodes	Edges	Node IDs
1	19.543	36	342	*IKBKB, EGFR, POLR2L, POLR2B, GSK3B, STUB1, PSMA2, HSP90AA1, UBA52, CASP1, UBB, ESR2, UBC, PSMA6, H2AC11, H3C12, H2BU1, H2AW, H3C8, H4C5, MUC1, EP300, HSP90AB1, HSPA1A, RB1, H4C2, KRAS, AKT1, TP53, FOXO3, PLK1, PSMA1, RPS27A, MGMT, BRCA1, ACTB*
2	5	7	15	*DNAJC5B, HSPA1L, HSPE1, DNAJB9, HSPH1, PTGES3, DNAJB12*
3	4.947	20	47	*POLR2L, UBE2E1, ATXN7, PSMA2, CASP1, UBA52, ESR2, UBB, CHD4, FOXO3, TRADD, PPP2R5C, H2BC11, PSMA6, FGF17, PSMA1, CSNK1A1, NSD2, NCL, MAP3K7*
4	4.5	5	9	*NEIL3, MSH2, CDC7, RAD54L, NUF2*
5	4.261	24	49	*BCLAF1, SDHD, SDHB, NUP93, ZC3H11A, SLC25A3, EXOSC6, NDUFA2, GCK, MPHOSPH6, PFKFB2, EXOSC7, NUP37, NUP155, CROT, ACAA1, PLIN1, CD36, THOC7, CYC1, THOC6, GATC, CCNC, TKTL2*
6	4	8	14	*ACTL6A, ATAD2, BRD3, ANLN, MND1, CKAP2L, BAZ1B, NCAPG*
7	3.714	8	13	*MRPL32, DDX24, DDX56, EBNA1BP2, MPHOSPH10, MRPL9, GNL1, ABCF1*
8	3.333	4	5	*ENO1, SAG, GNAQ, RHO*
9	3.333	4	5	*PYCARD, AIM2, IRF7, CASP5*
10	3.333	4	5	*CHST13, CHSY1, CSPG5, CHST14*
11	3.333	4	5	*C8G, C5, CPB2, TSPAN33*
12	3.143	8	11	*DDX6, IK, CPEB2, LSM5, PAPOLG, LSM2, ATXN2, UBAP2L*
13	3	3	3	*RASSF1, MGMT, CDKN2B*
14	3	3	3	*IL5, CD38, CD244*
15	3	3	3	*DTX3L, PARP9, IFIT2*
16	2.714	15	19	*CEP290, NR2E3, MYH7, HIBCH, ACADSB, MCCC1, PPP1R26, NEFL, ADSS2, KCNQ1, PPAT, MYOM2, IMPG2, ACSM3, IMPDH1*

**Table 4:** The evaluation metric results for five-fold cross-validation.

5-Fold	Accuracy	Precision	Recall	F1
K = 1	0.992	0.993	0.993	0.993
K = 2	1	1	1	1
K = 3	0.951	0.953	0.954	0.953
K = 4	0.984	0.984	0.983	0.984
K = 5	0.943	0.940	0.940	0.940
Avg	0.974	0.974	0.974	0.974

## Data Availability

Data were obtained from the Gene Expression Omnibus (GEO) database (https://www.ncbi.nlm.nih.gov/geo/) (accessed on 27 September 2023).
